# Withdrawn as duplicate: Differential Tolerance to Heat Stress of Young Leaves Compared to Mature Leaves of Whole Plants Relate to Differential Transcriptomes Involved in Metabolic Adaptations to Stress

**DOI:** 10.1093/aobpla/plac023

**Published:** 2022-05-25

## Abstract

This article has been withdrawn due to a publisher error that caused the article to be duplicated. The definitive version of this article is published under https://doi.org/10.1093/aobpla/plac024.

